# Design and Analysis of 5-DOF Compact Electromagnetic Levitation Actuator for Lens Control of Laser Cutting Machine

**DOI:** 10.3390/mi15050641

**Published:** 2024-05-10

**Authors:** Chuan Zhao, Qinwei Zhang, Wenzhe Pei, Junjie Jin, Feng Sun, Hongkui Zhang, Ran Zhou, Dongning Liu, Fangchao Xu, Xiaoyou Zhang, Lijian Yang

**Affiliations:** 1 School of Mechanical Engineering, Shenyang University of Technology, Shenyang 110870, China; zhaochuan@sut.edu.cn (C.Z.); zhangqinwei@smail.sut.edu.cn (Q.Z.); peiwenzhe@smail.sut.edu.cn (W.P.); sunfeng@sut.edu.cn (F.S.); zhouran@sut.edu.cn (R.Z.); dongningliu@sut.edu.cn (D.L.); xufangchao@sut.edu.cn (F.X.); 2 China Coal Technology & Engineering Group Shenyang Research Institute, Fushun 113122, China; 3 Department of Mechanical Engineering, Nippon Institute of Technology, Saitama 345-8501, Japan; zhang.xiaoyou@nit.ac.jp; 4 School of Information Science and Engineering, Shenyang University of Technology, Shenyang 110870, China

**Keywords:** electromagnetic levitation actuator, laser cutting machine, PID control, coordinate transform, magnetic field analysis

## Abstract

In laser beam processing, the angle or offset between the auxiliary gas and the laser beam axis have been proved to be two new process optimization parameters for improving cutting speed and quality. However, a traditional electromechanical actuator cannot achieve high-speed and high-precision motion control with a compact structure. This paper proposes a magnetic levitation actuator which could realize the 5-DOF motion control of a lens using six groups of differential electromagnets. At first, the nonlinear characteristic of a magnetic driving force was analyzed by establishing an analytical model and finite element calculation. Then, the dynamic model of the magnetic levitation actuator was established using the Taylor series. And the mathematical relationship between the detected distance and five-degree-of-freedom was determined. Next, the centralized control system based on PID control was designed. Finally, a driving test was carried out to verify the five-degrees-of-freedom motion of the proposed electromagnetic levitation actuator. The results show it can achieve a stable levitation and precision positioning with a desired command motion. It also proves that the proposed magnetic levitation actuator has the potential application in an off-axis laser cutting machine tool.

## 1. Introduction

With the rapid development of the aerospace, automobile and semiconductor industries, the processing difficulty of key parts with complex structures and high-hardness materials is gradually increasing. Improving efficiency under the premise of ensuring machining accuracy and indicating quality has become an important challenge for advanced manufacturing technology. Laser cutting has been widely applied due to its characteristics of high precision, high efficiency, small thermal deformation, low noise, strong flexibility and small thermal deformation.

In laser cutting processing, the processing method of coaxial laser beam and auxiliary gas nozzle has basically been adopted, and the research on the laser cutting process is mostly focused on the analysis of the nozzle auxiliary gas flow field and structural improvement. The research on traditional laser cutting technology mostly focuses on the improvement of the nozzle structure and the analysis of the auxiliary gas flow field [1,2,3]. Its processing quality and efficiency are affected by laser power, pulse frequency, gas pressure, feed rate, defocusing amount, sheet properties and thickness. [4] proposed a new process optimization scheme, which can effectively improve the processing efficiency of the laser cutting machine and the processing quality of the kerf by making the blowing direction of the auxiliary gas and the laser beam form a certain angle. However, this different axis will also lead to a different processing efficiency and quality of the laser cutting machine in each processing feed direction, which will seriously affect the processing accuracy. In order to solve the influence of this inconsistency, a high-speed, high-precision and compact driver must be used to control the lens to ensure the position relationship between the laser beam and the auxiliary airflow in real time in different feed directions.

The electromechanical actuators cannot achieve a multi-degree of freedom driving motion with a compact structure due to the existence of the contact transmission mechanism. The magnetic levitation technology can realize force regulation without contact. In a multi-DOF driving system, the magnetic levitation technology is highly favorable for the following characteristics: (i) a compact structure as the driving force and torque are generated by an integrated maglev actuator; (ii) a high motion precision with active control and positioning errors compensation; (iii) the elimination of vibration noise owing to its non-contact advantage [5,6]

The maglev actuators can be categorized into Lorentz actuators and reluctance actuators. Dyck [7] developed a 6-DOF magnetically levitated rotary table for micro-positioning. This stage uses a combination of four Lorentz-force magnetically levitated linear motors to achieve an unlimited rotation motion range about the vertical axis. Heyman [8] designed a Lorentz force-based magnetically levitated stage which can achieve a 10 mm stroke in all XYZ directions. Gloess [9] presented a magnetically levitated hub actuator. This stage prototype can generate thrust forces in the X and Y directions of up to 200 N. Zhang [10] proposed a MagTable which consists of a planar array of square coils and a permanent magnet type carrier. The maximum levitation height of the carrier is 30 mm within a 400 mm × 200 mm horizontal translation range. Huang [11,12] proposed a min–max model predictive control (MPC) method of planar motors, which can achieve robust precision position tracking. The Lorentz actuators can achieve a long stroke with nanometer positioning. However, it is difficult to achieve laser lens driving by Lorentz force due to a large volume caused by the PM array or armature winding. 

The magnetic bearing is a typical application of reluctance levitation technology [13]. In order to support the rotor of rotating machinery, the five-degree-of-freedom magnetic bearing system often adopts a distributed scheme [14]. Two radial magnetic bearings realize the three-degree-of-freedom control on both sides of the rotor, and a thrust magnetic bearing is used to move the rotor axially [15,16]. Masahiro [17] designed a maglev motor with a 5-DOF active control. The movable ranges of the rotor in the axial and radial direction are restricted to ±0.3 mm and ±0.5 mm, respectively. Luan [18] and Zhang [19] designed a controllable magnetic levitation actuator for an EDM machine tool to improve the stability of the inter pole voltage, hence the machining speed increases to 3.925 μs. Dongjue He [20,21] designed a novel air core coil type electro-magnetic driving unit to actuate the lens holder, which can achieve a range of ±5 mm with a tracking error of less than 12 μm and a bandwidth of more than 100 Hz in the axial direction. However, the above magnetic levitation driver has a large volume, a large mover mass, and a large motion inertia, resulting in slow control accuracy and response speed.

Therefore, this project proposes a five-degree-of-freedom magnetic levitation driver with a compact structure, which adopts six sets of differential electromagnets to achieve five-degree-of-freedom motion. The dynamic model of the five-degree-of-freedom magnetic levitation drive device is established. The characteristics of the electromagnetic force of the linearized model are analyzed, and the mathematical model between the sensor and the actual displacement of the suspension platform is derived. The PID control is used to verify the five-degrees-of-freedom motion of the system, and the displacement response and position control characteristics of the system are analyzed. In the control of each degree of freedom motion, it can achieve a stable suspension and good tracking effect of the desired command, and has a certain robustness.

## 2. 5-DOF Magnetic Levitation Driver

### 2.1. Magnetic Levitation Driver Function

Traditional laser cutting technology requires the laser beam to be coaxial with the auxiliary gas in order to ensure the consistency of the cutting quality in the processing feed direction. A review of the literature reveals that the eccentricity of the laser beam with the auxiliary gas improves the quality and efficiency of the process. This paper proposes a magnetic levitation drive for a five-degree-of-freedom laser light path. It realizes high cutting quality and efficiency, non-contact 5-DOF motion, reduced friction and improved system response characteristics. The ranges of the magnetic levitation actuator in five-degrees-of-freedom are, respectively, 0.05 mm in axial range, 0.1 mm in radial range, 0.001 rad in α direction, and 0.001 rad in β direction.

**Figure 1:**
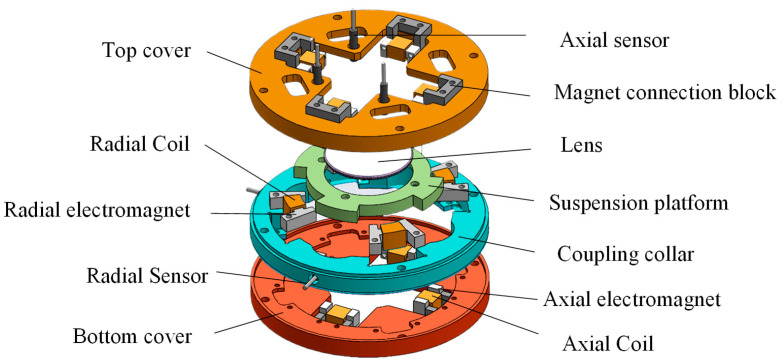
Structural diagram of a 5-DOF magnetic levitation actuator.

The magnetic levitation drive is shown in Figure 1. The drive as a whole consists of a top cover, a bottom cover and a connecting ring in the center. There is an aluminum ring in the middle of the suspended platform to place the laser lens, and the control of the suspended platform realizes the control of the laser lens to achieve the purpose of controlling the laser light path and the off-axis effect. Among them, four sets of axial differential electromagnets are evenly distributed in the upper and lower covers, which can make the floating platform realize the *z*-axis direction and α, β direction movement; the connecting ring in the middle part is likewise evenly distributed with two sets of radial differential solenoids in 45° relation to the axial solenoids, which can realize the movement of the floating platform in the direction of the X and Y axes.

### 2.2. 5-DOF Magnetic Levitation Actuator Principle of Operation

**Figure 2:**
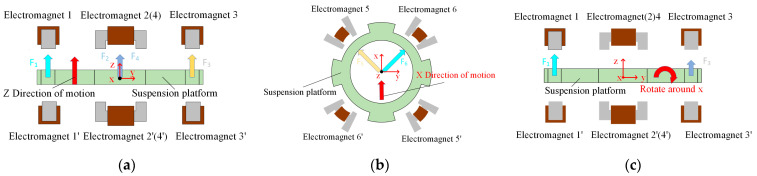
Motion control principle of suspended platform: (**a**) z direction, (**b**) x direction, (**c**) α direction.

**Figure 3:**
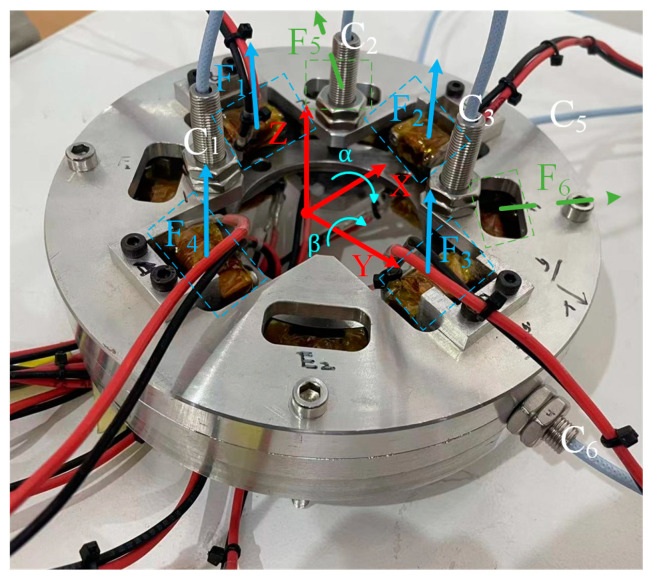
The 5-DOF magnetic levitation drive.

The 5-DOF motion of the magnetic levitation actuator is controlled by the electromagnetic force of a differential electromagnet. In the experiments, the translations along the x and y directions are similar, as are the α and β direction rotations. Therefore, in this paper, only the principles of translation in the z and x directions and the rotation in the α direction are presented. As shown in Figure 2, there are four sets of differential electromagnets labeled 1 (1′), 2 (2′), 3 (3′), and 4 (4′) in the vertical direction; and there are two sets of differential electromagnets 5 (5′) and 6 (6′) in the horizontal direction in a diagonal arrangement. In Figure 2a, the combined force generated by the four sets of differential electromagnets in the vertical direction is in the same direction as the axis, thereby driving the suspended platform in that direction. In Figure 2b, the electromagnets 5 (5′) and electromagnets 6 (6′) generate a combined force pointing in the positive direction of the *X*-axis, thereby driving the suspended platform along that direction. In Figure 2c, the torque generated by electromagnet 1 (1′) is in the opposite direction to the movement generated by electromagnet 3 (3′), thereby driving the levitated platform to rotate in that direction. The experimental setup is shown in Figure 3.

## 3. Analysis of Magnetic Field Characteristics of Magnetic Drive Platform

### 3.1. Axial Single Electromagnetic Force Analysis

First, the magnetic field finite element software is used to simulate the five-degree-of-freedom magnetic levitation drive model. The core magnetic material is set as silicon steel, the levitation platform material is set as Q235, the coil material is set as copper, and other materials are set as aluminum. In order to ensure the accuracy of the calculation results, the model adopts adaptive mesh and refines the key parts such as arc air gap and levitation air gap. The levitation platform is set as the force object, the variable is set as the control current, and the range of the parameterized scan is 0–1.5 A, with a step size of 0.1 A.

**Figure 4:**
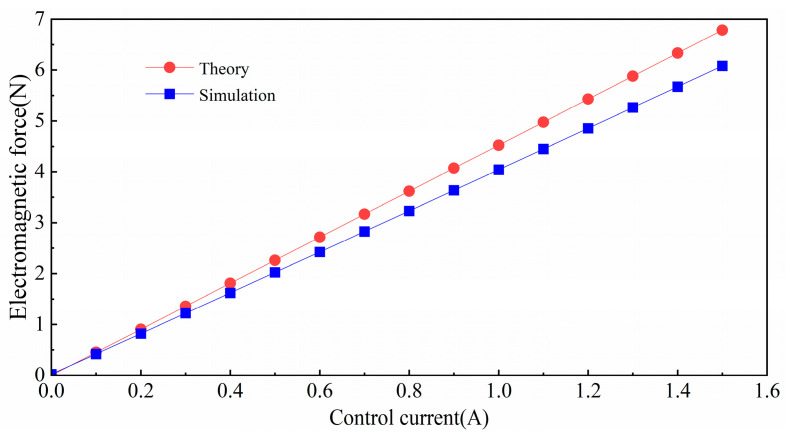
Comparison of theoretical and simulated electromagnetic forces.

Then, the simulated electromagnetic force is compared with the theoretical value calculated by the simplified model of differential electromagnetic force Equation (1) using the simulated electromagnetic force, and the change in the electromagnetic force with the excitation current at the axial equilibrium position is compared and analyzed, as shown in Figure 4.

(1) $$F=F_{1}-F_{2}=\frac{\mu_{0}N^{2}A{\left(i+i_{0}\right)}^{2}}{4{\left(d_{0}-z\right)}^{2}}-\frac{\mu_{0}N^{2}A{\left(i-i_{0}\right)}^{2}}{4{\left(d_{0}+z\right)}^{2}}$$

In the above equation, N is the number of turns of coil required to wind the solenoid, A is the cross-sectional area of the magnetic circuit air gap, i is the current in the coil of the solenoid, $i_{0}$ is the bias current, and $d_{0}$ is the balance air gap.

The comparison between simulation and theoretical calculation shows that the simulated electromagnetic force has the same trend as the theoretical electromagnetic force. At the maximum control current of 1.5 A, the maximum error between theory and simulation is 10%, which meets the design requirements and indicates that the structural design is reasonable. The results also show that the magnetic force of 3.64 N at a control current of 0.9 A satisfies the experimental requirements for a levitated platform under current differential control. The structural parameters and solenoid parameters of which are simulated are shown in Table 1.

### 3.2. Equilibrium Position Electromagnetic Force Analysis

**Figure 5:**
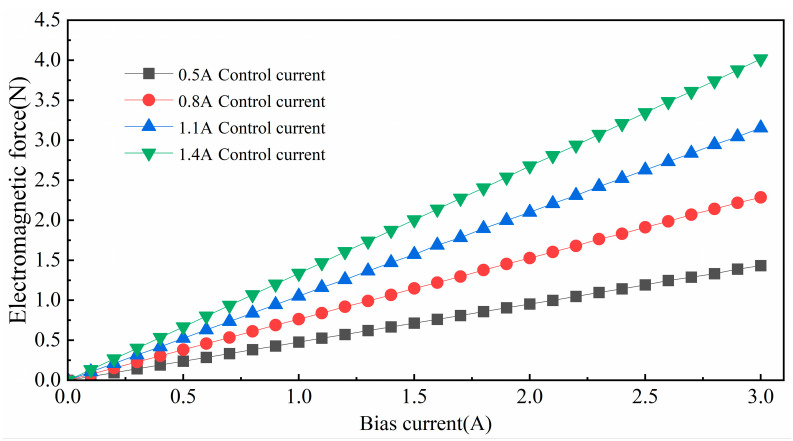
Variation in electromagnetic force with different bias currents.

The variation in the axial electromagnetic force with the bias current under different control currents is analyzed using the finite element method and the simulation results are shown in Figure 5.

The analysis results show the change in electromagnetic force at the equilibrium position with different bias currents; different control currents are selected to observe the change in electromagnetic force and it can be seen from the simulation that the electromagnetic force has a good linear relationship at the equilibrium position.

**Figure 6:**
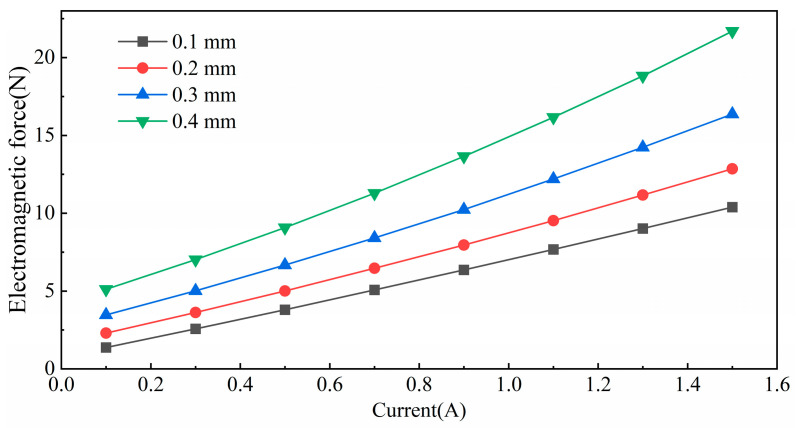
Variation in electromagnetic force with control current under different axial displacements.

Furthermore, the impact of axial displacement at the equilibrium position and the selection of the control current on the performance of the axial electromagnetic force was analyzed. The equilibrium position was set at 0 mm, and the variation in the axial electromagnetic force with the control current was examined as the axial displacement was varied from 0.1 mm to 0.4 mm. The results of this analysis are presented in Figure 6.

The results show that the slope of the curve remains basically unchanged when the displacement is less than 0.4 mm; there is a significant increase in the slope and the nonlinear characteristics of the electromagnetic force begin to appear.

In the design of the 5-DOF magnetic levitation actuator, in order to ensure stability during operation, the levitation platform should have a good linear workspace at the steady state operating point, which is expected by the design. So, the electromagnetic force is simulated at different bias currents and at bias current 1.5 A and the electromagnetic force has a good linear space. Additionally, the variation in the electromagnetic force with the control current in the displacement case is also simulated. The simulation results show that the electromagnetic force within the equilibrium position range exhibits good linear characteristics. Consequently, the designed 5-DOF maglev actuator demonstrates a favorable linear workspace at the equilibrium position.

### 3.3. Magnetic Field Analysis

**Figure 7:**
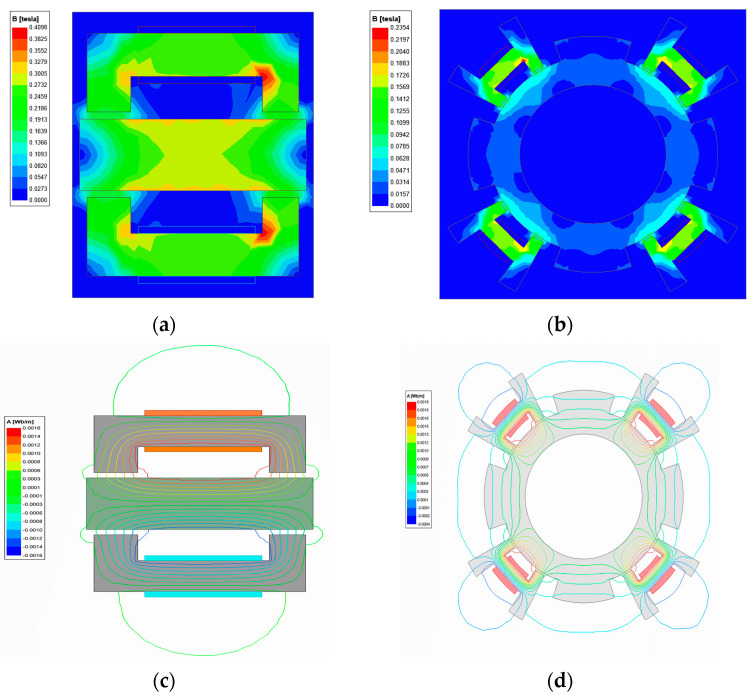
Magnetic field simulation of a 5-DOF magnetic levitation actuator. (**a**) Axial magnetic field intensity. (**b**) Radial magnetic field intensity. (**c**) Axial magnetic circuit. (**d**) Radial magnetic circuit.

Figure 7 depicts the magnetic field simulation in the axial and radial directions, respectively. To facilitate the clear observation of the axial magnetic field simulation, a set of electromagnet simulations is utilized. The five-degree-of-freedom magnetic drive platform model is simulated using simulation software, with a maximum current of 3 A applied to the axial and radial electromagnets for excitation, respectively. The simulation results indicate that the magnetic field strength and magnetic circuit of the levitated platform are consistent with theoretical expectations. Furthermore, it is demonstrated that the structural design of the five-degree-of-freedom magnetic levitation actuator avoids magnetic leakage.

## 4. Mathematical Modeling of a 5-DOF Maglev Actuator

### 4.1. Sensor Coordinate Transformation for Magnetic Levitation Actuators

When performing levitation experiments with a five-degree-of-freedom magnetic levitation actuator, it is necessary to transform the coordinates of the sensor and the degrees of freedom. According to the working principle of a differential electromagnet, by controlling the size of the control current of the electromagnet, the size of the electromagnetic force of the levitation platform can be changed to realize the movement of five-degrees-of-freedom. The axial sensors are C_1_, C_2_ and C_3_, and the radial sensors are C_5_ and C_6_. When the platform moves in the x and y directions, it can be measured directly without solving the derivation. When the platform moves in other degrees of freedom, the offset detected using the sensors for each degree of freedom is not the actual controlled offset and needs to be solved. This discrepancy is caused by the fact that the centerline of the sensor is not in the same line as the centerline of the magnetic poles. Where the relationship between the measurement signal of the sensor and the degrees of freedom is shown in Equation (2):

(2) $$\left\{\begin{array}{c} z=\frac{1}{3}\left(d_{1}+d_{2}+d_{3}\right)\\ \alpha =\frac{d_{2}-d_{1}}{2L_{1}\sin\theta }\\ \beta =\frac{d_{3}-d_{2}}{2L_{1}\sin\theta }\\ x=d_{5}\\ y=d_{6} \end{array}\right.$$

Rewrite in matrix form:

(3) $$\left[\begin{array}{l} z\\ \alpha \\ \beta \\ x\\ y \end{array}\right]=N\left[\begin{array}{l} d_{1}\\ d_{2}\\ d_{3}\\ d_{5}\\ d_{6} \end{array}\right]$$

N is the coordinate transformation matrix of the sensor and the degrees of freedom:

$$N=\left[\begin{array}{ccccc} \frac{1}{3} & \frac{1}{3} & \frac{1}{3} & 0 & 0\\ \frac{1}{-2L_{1}\sin\theta } & \frac{1}{-2L_{1}\sin\theta } & 0 & 0 & 0\\ 0 & \frac{1}{-2L_{1}\sin\theta } & \frac{1}{2L_{1}\sin\theta } & 0 & 0\\ 0 & 0 & 0 & 1 & 0\\ 0 & 0 & 0 & 0 & 1 \end{array}\right]$$

### 4.2. Modeling of Magnetic Levitation Drive Systems

**Figure 8:**
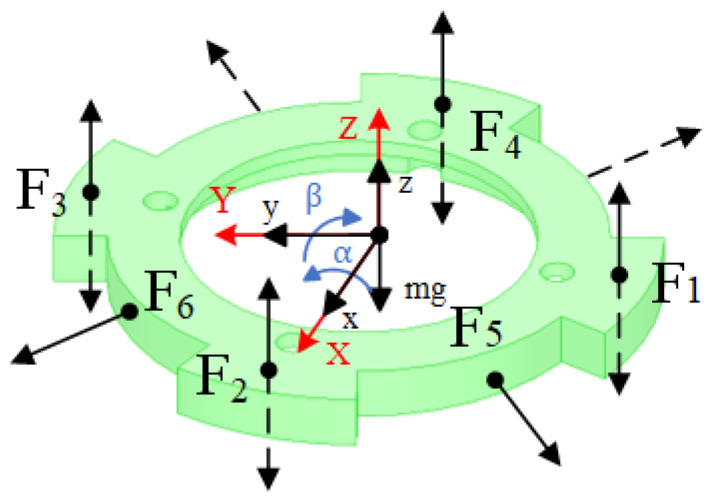
Force analysis of suspension platform.

From the 5-DOF magnetic levitation actuator system, the force analysis of the levitated platform is shown in Figure 8. F is the magnetic force of the electromagnet. z, α, and β are the displacement of the suspended platform along the *Z*-axis, the angle of rotation around the X-axis, and the angle of rotation around the Y-axis, respectively. $L_{1}$, $L_{2}$, and $L_{3}$ are the distances between the sensor, axial electromagnet, and radial electromagnet, respectively. θ is the angle between the sensor and the X-axis. m is the mass of the suspended platform.

The dynamics of a 5-DOF magnetic levitation actuator is modeled according to the Lagrange equations:

(4) $$\begin{array}{c} m\ddot{x}=\frac{\sqrt{2}}{2}(F_{5}+F_{6})-c_{x}\dot{x}+f_{x}\\ m\ddot{y}=\frac{\sqrt{2}}{2}(F_{5}-F_{6})-c_{y}\dot{y}+f_{y}\\ m\ddot{z}=F_{1}+F_{2}+F_{3}+F_{4}-c_{z}\dot{z}-mg+f_{z}\\ J_{\alpha }\ddot{\alpha }=F_{1}L_{2}-F_{3}L_{2}-c_{\alpha }\dot{\alpha }+T_{\alpha }\\ J_{\beta }\ddot{\beta }=F_{4}L_{2}-F_{2}L_{2}-c_{\beta }\dot{\beta }+T_{\beta } \end{array}$$

where $F_{1}$ to $F_{6}$ are the magnetic forces of each of the six electromagnets. $c_{z}$, $c_{x}$, $c_{y}$, $c_{\alpha }$, $c_{\beta }$ are the damping coefficients for the Z-axis, X-axis, Y-axis, rotation around the X-axis, and rotation around the Y-axis, respectively. $f_{z}$, $f_{x}$, $f_{y}$, $T_{\alpha }$, $T_{\beta }$ are the perturbation forces in the Z-axis, X-axis, Y-axis, rotation around the X-axis, and rotation around the Y-axis, respectively.

(5) $$\begin{array}{l} F_{n}=k_{i}i_{n}+k_{d}d_{n},n=1,2,3,4\\ F_{m}=k_{i}^{\prime }i_{m}+k_{d}^{\prime }d_{m},m=5,6 \end{array}$$

(6) $$k_{i}=\frac{4Ki_{0}}{{d_{0}}^{2}},k_{d}=\frac{4K{i_{0}}^{2}}{{d_{0}}^{3}}$$

The linear differential equation of the system is obtained, as shown in Equation (7), by applying the linearization Equation (5) to Equation (4).

(7) $$\begin{array}{c} m\ddot{x}=\frac{\sqrt{2}}{2}k_{i}^{\prime }(i_{5}+i_{6})+\frac{\sqrt{2}}{2}k_{d}^{\prime }(d_{5}+d_{6})-c_{x}\dot{x}+f_{x}\\ m\ddot{y}=\frac{\sqrt{2}}{2}k_{i}^{\prime }(i_{5}-i_{6})+\frac{\sqrt{2}}{2}k_{d}^{\prime }(d_{5}-d_{6})-c_{y}\dot{y}+f_{y}\\ m\ddot{z}=k_{i}(i_{1}+i_{2}+i_{3}+i_{4})+k_{d}(d_{1}+d_{2}+d_{3}+d_{4})-c_{z}\dot{z}+f_{z}\\ J_{\alpha }\ddot{\alpha }=k_{i}L_{2}(i_{1}-i_{3})+k_{d}L_{2}(d_{1}-d_{3})-c_{\alpha }\dot{\alpha }+T_{\alpha }\\ J_{\beta }\ddot{\beta }=k_{i}L_{2}(i_{4}-i_{2})+k_{d}L_{2}(d_{4}-d_{2})-c_{\beta }\dot{\beta }+T_{\beta } \end{array}$$

Organized in matrix form:

(8) $$M\left[\begin{array}{c} \ddot{x}\\ \ddot{y}\\ \ddot{z}\\ \ddot{\alpha }\\ \ddot{\beta } \end{array}\right]=K_{I}\left[\begin{array}{c} i_{1}\\ i_{2}\\ i_{3}\\ i_{4}\\ i_{5}\\ i_{6} \end{array}\right]+K_{D}\left[\begin{array}{c} d_{1}\\ d_{2}\\ d_{3}\\ d_{4}\\ d_{5}\\ d_{6} \end{array}\right]-C\left[\begin{array}{c} \dot{x}\\ \dot{y}\\ \dot{z}\\ \dot{\alpha }\\ \dot{\beta } \end{array}\right]+\left[\begin{array}{c} f_{x}\\ f_{y}\\ f_{z}\\ T_{\alpha }\\ T_{\beta } \end{array}\right]$$

M is the inertial matrix of the suspension platform, $K_{I}$ is the current coefficient matrix, $K_{D}$ is the displacement coefficient matrix, and C is the damping coefficient matrix.

$$\begin{array}{ccc} M & = & \left[\begin{array}{ccccc} m & 0 & 0 & 0 & 0\\ 0 & m & 0 & 0 & 0\\ 0 & 0 & m & 0 & 0\\ 0 & 0 & 0 & J_{\alpha } & 0\\ 0 & 0 & 0 & 0 & J_{\beta } \end{array}\right]\\ K_{I} & = & \left[\begin{array}{cccccc} 0 & 0 & 0 & 0 & \frac{\sqrt{2}}{2}k_{i}^{\prime } & \frac{\sqrt{2}}{2}k_{i}^{\prime }\\ 0 & 0 & 0 & 0 & \frac{\sqrt{2}}{2}k_{i}^{\prime } & -\frac{\sqrt{2}}{2}k_{i}^{\prime }\\ k_{i} & k_{i} & k_{i} & k_{i} & 0 & 0\\ k_{i}L_{2} & 0 & -k_{i}L_{2} & 0 & 0 & 0\\ 0 & -k_{i}L_{2} & 0 & k_{i}L_{2} & 0 & 0 \end{array}\right]\\ K_{D} & = & \left[\begin{array}{cccccc} 0 & 0 & 0 & 0 & \frac{\sqrt{2}}{2}k_{d}^{\prime } & \frac{\sqrt{2}}{2}k_{d}^{\prime }\\ 0 & 0 & 0 & 0 & \frac{\sqrt{2}}{2}k_{d}^{\prime } & -\frac{\sqrt{2}}{2}k_{d}^{\prime }\\ k_{d} & k_{d} & k_{d} & k_{d} & 0 & 0\\ k_{d}L_{2} & 0 & -k_{d}L_{2} & 0 & 0 & 0\\ 0 & -k_{d}L_{2} & 0 & k_{d}L_{2} & 0 & 0 \end{array}\right]\\ C & = & \left[\begin{array}{ccccc} c_{x} & 0 & 0 & 0 & 0\\ 0 & c_{y} & 0 & 0 & 0\\ 0 & 0 & c_{z} & 0 & 0\\ 0 & 0 & 0 & c_{\alpha } & 0\\ 0 & 0 & 0 & 0 & c_{\beta } \end{array}\right] \end{array}$$

The coordinate transformations of magnetic pole displacements and degrees of freedom:

(9) $$\left\{\begin{array}{l} x=\frac{\sqrt{2}}{4}d_{5}+\frac{\sqrt{2}}{4}d_{6}\\ y=\frac{\sqrt{2}}{4}d_{5}-\frac{\sqrt{2}}{4}d_{6}\\ z=\frac{1}{4}(d_{1}+d_{2}+d_{3}+d_{4})\\ \alpha =\frac{1}{2L_{2}}(d_{1}-d_{3})\\ \beta =\frac{1}{2L_{2}}(d_{4}-d_{2}) \end{array}\right.$$

Organized in matrix form:

(10) $$\left[\begin{array}{c} x\\ y\\ z\\ \alpha \\ \beta \end{array}\right]=N_{1}\left[\begin{array}{l} d_{1}\\ d_{2}\\ d_{3}\\ d_{4}\\ d_{5}\\ d_{6} \end{array}\right]$$

$N_{1}$ is the distribution matrix

$$N_{1}=\left[\begin{array}{cccccc} 0 & 0 & 0 & 0 & \frac{\sqrt{2}}{4} & \frac{\sqrt{2}}{4}\\ 0 & 0 & 0 & 0 & \frac{\sqrt{2}}{4} & -\frac{\sqrt{2}}{4}\\ \frac{1}{4} & \frac{1}{4} & \frac{1}{4} & \frac{1}{4} & 0 & 0\\ \frac{1}{2L_{2}} & 0 & -\frac{1}{2L_{2}} & 0 & 0 & 0\\ 0 & -\frac{1}{2L_{2}} & 0 & \frac{1}{2L_{2}} & 0 & 0 \end{array}\right]$$

Organize the matrix:

(11) $$\left[\begin{array}{l} d_{1}\\ d_{2}\\ d_{3}\\ d_{4}\\ d_{5}\\ d_{6} \end{array}\right]=N_{2}\left[\begin{array}{c} x\\ y\\ z\\ \alpha \\ \beta \end{array}\right]$$

After organizing the matrix, a system dynamics model is obtained, with the model parameters shown in Table 2. The model can be expressed as follows.

(12) $$M\left[\begin{array}{c} \ddot{x}\\ \ddot{y}\\ \ddot{z}\\ \ddot{\alpha }\\ \ddot{\beta } \end{array}\right]=K_{I}N_{2}\left[\begin{array}{c} i_{x}\\ i_{y}\\ i_{z}\\ i_{\alpha }\\ i_{\beta } \end{array}\right]+K_{D}N_{2}\left[\begin{array}{c} x\\ y\\ z\\ \alpha \\ \beta \end{array}\right]-C\left[\begin{array}{c} \dot{x}\\ \dot{y}\\ \dot{z}\\ \dot{\alpha }\\ \dot{\beta } \end{array}\right]+\left[\begin{array}{c} f_{x}\\ f_{y}\\ f_{z}\\ T_{\alpha }\\ T_{\beta } \end{array}\right]$$

## 5. Levitation Experiments with a 5-DOF Magnetic Levitation Actuator

### 5.1. Centralized Control Strategy for Magnetic Levitation Drives

**Figure 9:**
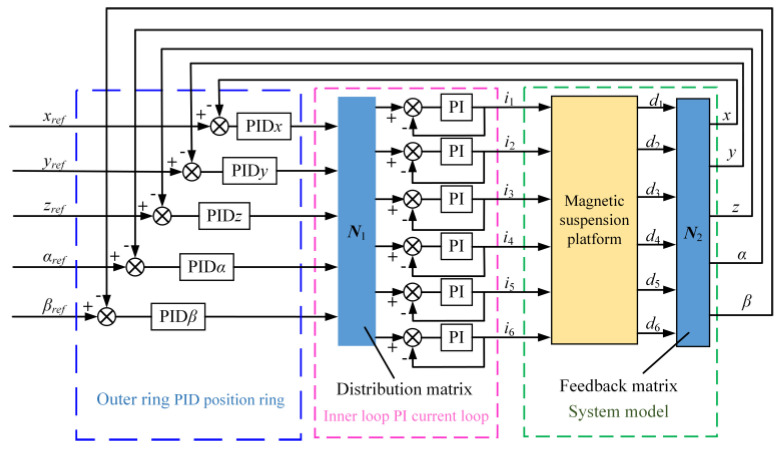
The block diagram of five-degree-of-freedom centralized control system.

The system model of the five-degree-of-freedom magnetic levitation actuator comprises two parts: the model for the five-degree-of-freedom motion and the model for each group of electromagnets. The closed-loop control system adopts a series-level control structure. The outer loop controls the five-degrees-of-freedom of the platform and utilizes a PID control law, serving as the primary regulation loop of the control system. The inner loop controls the current of the electromagnet and adopts a PI control. A platform PID control system is established, as depicted in Figure 9. The reference inputs for the platform’s five-degrees-of-freedom ($x_{ref}$, $y_{ref}$, $z_{ref}$ $\alpha_{ref}$, $\beta_{ref}$) are set, and the outer loop PID controller is adjusted based on the error. The inner loop employs a PI current loop to ensure that the output current quickly tracks the output voltage of the levitation controller within a certain frequency range, thereby enhancing the current response of the magnetic levitation drive and achieving control over the five-degrees-of-freedom motion.

### 5.2. Experimental System Composition

**Figure 10:**
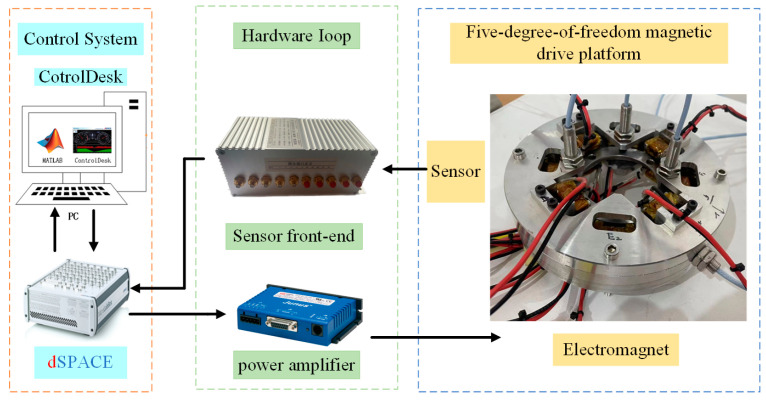
Experimental system.

The experimental system for the five-degree-of-freedom magnetic levitation actuator is illustrated in Figure 10, comprising the prototype, hardware equipment, and control system. The control system is based on the DS1202 control board from dSPACE, with ControlDesk 7.6 software toolkits installed on the host computer. The hardware circuit utilizes drivers, while air gap detection employs eddy current displacement sensing technology from Zhuzhou Liulingba Technology and Science Co., Ltd. (Zhuzhou, China). The detection range is 0.65 mm to 2.65 mm and the analog output voltage range is 0 V to 10 V.

### 5.3. Magnetic Levitation Drive Experiment

In this system, the sensor displacement needs to be determined. The sensor measurement is resolved for the z, α and β degrees of freedom. When the sensor measurement is 0 mm, it indicates that the levitated platform is attracted by the electromagnet; when the air gap is measured to be 1.12 mm, the levitated platform reaches its lowest point.

**Figure 11:**
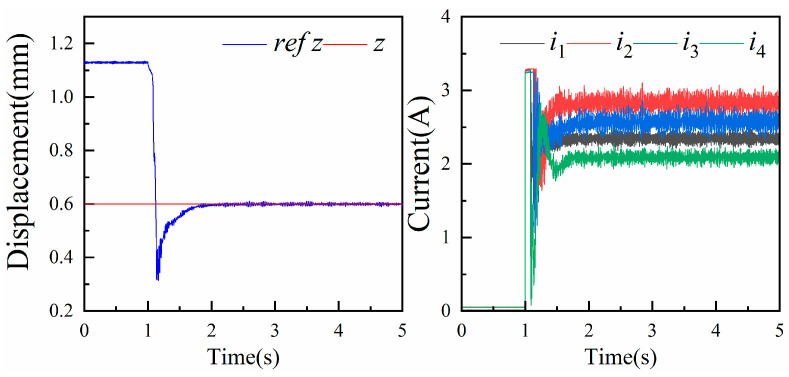
Floatation experiments and current changes.

We set the levitated platform to float at 0.6 mm, midway within the air gap. To stabilize the levitation platform at this height, the magnitude of the current is adjusted so that the electromagnetic force on the levitation platform equals the force of gravity. The changes in the levitation displacement and current are demonstrated in Figure 11. This lays the foundation for subsequent translational and rotational experiments. Additionally, the levitation experiment can verify whether the magnetic levitation actuator can achieve a stable levitation state at the midpoint position. The results indicate that the system can achieve a stable levitation at 0.6 mm after levitation.

**Figure 12:**
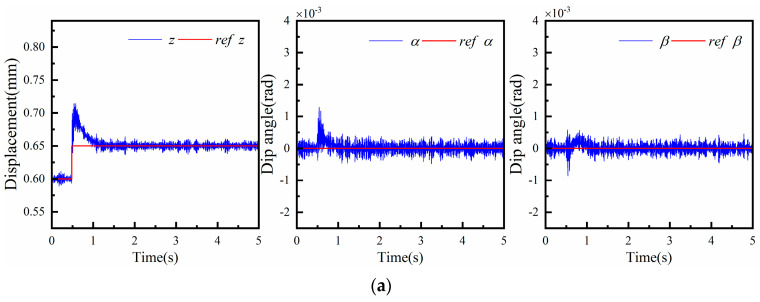

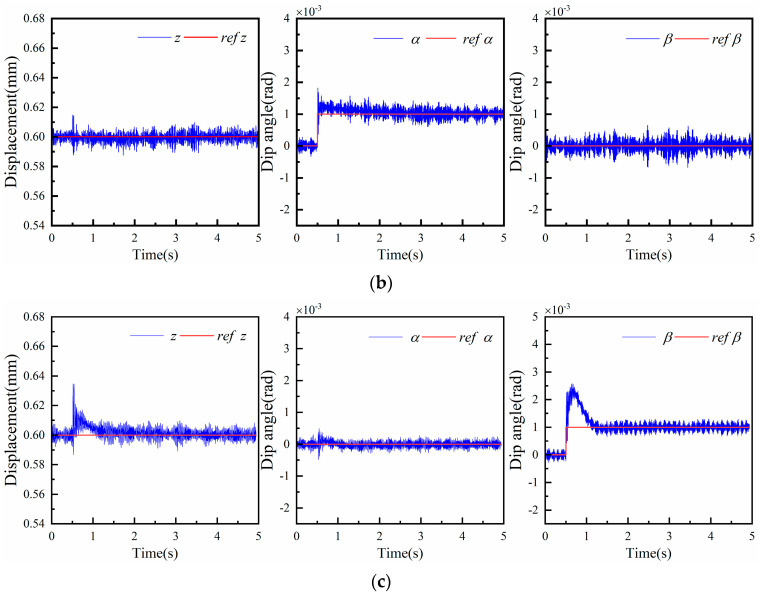
The z, α, β step response of PID control.(**a**) z direction; (**b**) α direction; (**c**) β direction.

When the levitation platform is stably suspended, step signals are applied to the z degree of freedom, α degree of freedom, and β degree of freedom in turn, and the experimental results are shown in Figure 12. In the initial state, the platform is in a stable suspension position, corresponding to an air gap length of 0.6 mm, and the platform deflection angles α and β are both zero radians. As shown in Figure 12a, a 0.05 mm step signal is input to the z degree of freedom at 0.5 s. The platform reaches a new levitation state after about 0.7 s, at which time the platform levitates with an air gap of 0.65 mm. Additionally, the platform deflection angles α and β remain zero radians during the adjustment process, and the z degree of freedom step input does not interfere with the platform rotational degrees of freedom. As shown in Figure 12b, a step signal of 0.001 rad is applied to the α degree of freedom at 0.5 s, and the system stabilizes after about 1.1 s. The system is then stabilized. The z degree of freedom and the B degree of freedom remain in the same state as before the step is applied; the suspension height is 0.6 mm. As shown in Figure 12c, a step signal of 0.001 rad is applied to the β degree of freedom at 0.5 s, and the system stabilizes after about 0.6 s. The system is then stabilized. At this point, the three degrees of freedom changes in the platform relative to the initial state after stabilizing the suspension are 0.6 mm, 0.001 rad, and 0.001 rad, respectively.

**Figure 13:**
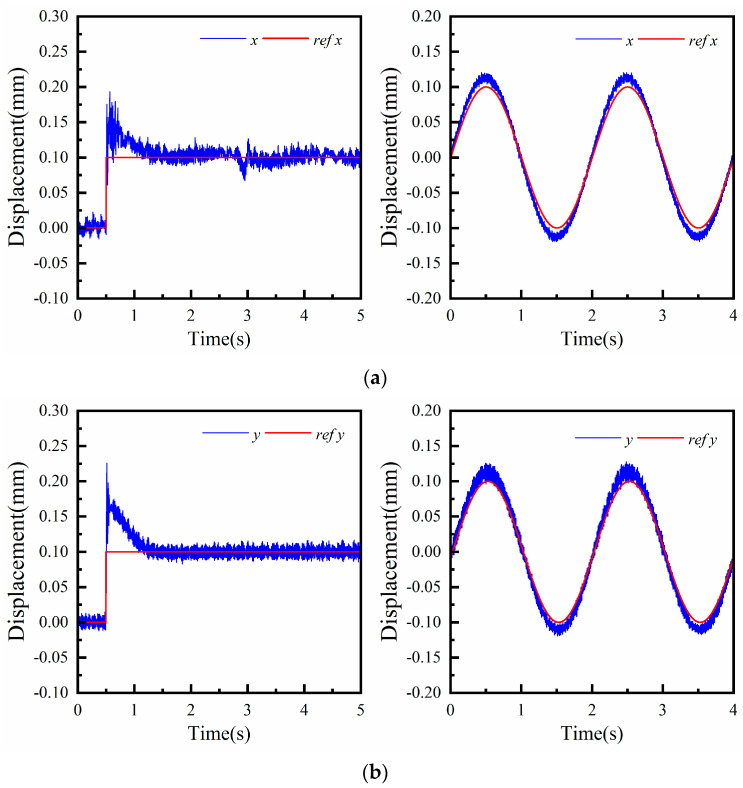
y step response of PID control. (**a**) x direction. (**b**) y direction.

Similarly, when the platform is stably levitated, the step and sinusoidal signals are applied to the x and y degrees of freedom in turn, and the experimental results are shown in Figure 13. In the initial state, the platform is in a stable levitation position, with the air gap length corresponding to z being 0.6 mm, and the platform deflection angles A and B are both zero radians. As shown in Figure 13a, a step signal of 0.1 mm is input to the x degree of freedom at 0.5 s, and the platform reaches a new levitation state after about 0.8 s, at which point the platform has an air gap of 0.6 mm. The x degree of freedom is also tracked. The tracking characteristics of the x degree of freedom are further analyzed by applying a sinusoidal signal with a frequency of 0.5 Hz. The trajectory is tracked with an amplitude ratio of 1.12 and a phase difference of 2.9°. Additionally, the other degrees of freedom of the suspended platform remain at zero during the adjustment process. As shown in Figure 13b, a step signal of 0.1 mm is applied to the y degree of freedom at 0.5 s, and the system stabilizes after about 0.8 s. A sinusoidal signal with a frequency of 0.5 Hz and an amplitude of 0.1 mm is applied, and the trajectory is tracked with an amplitude ratio of 1.17 and a phase difference of 0.2°.

During the above experiments, the levitation platform is able to maintain stable levitation after applying a step to each degree of freedom. It can be seen that the structure of the 5-DOF magnetic levitation actuator is reasonably designed.

## 6. Discussion and Recommendations

The experimental results of the five-degree-of-freedom magnetic levitation actuator are given in Figure 12 and Figure 13 for the step response and trajectory tracking in different degrees of freedom. The main reason for the large overshoot of each step response is that the magnetic levitation system is a non-damped system, and the overshoot and response time for PID control are in some contradiction; a small overshoot will inevitably be sacrificed for a certain response time. Other reasons may be that the selection of PID parameters is not optimal. The comparison of the magnetic levitation drive designed in this paper with the published results shows that the experimental results are somewhat deficient. Both the overshoot and the response time are not at the expected level. At the same time, there are power loss and temperature problems. However, the five-degree-of-freedom magnetic levitation actuator with the six group differential control proposed in this paper has the advantages of a compact structure and a certain robustness in five-degree-of-freedom motion. We believe that through subsequent optimization, this design will show higher application potential.

## 7. Conclusions

In this paper, a 5-DOF compact electromagnetic levitation actuator for lens control was designed. The nonlinear characteristics of the magnetic driving force were analyzed by establishing an analytical model and conducting finite element calculations. Next, we established the dynamic model of the magnetic levitation actuator. A centralized control system based on the PID control was designed, and driving experiments were conducted to verify the motion in five-degrees-of-freedom. The main conclusions are as follows:

1. The five-degree-of-freedom magnetic levitation actuator exhibits a positive correlation between the electromagnetic force and the control current within the range of 0 to 1.5 A. The maximum output electromagnetic force reaches 6.1 N. Specifically, at a control current of 0.9 A, the electromagnetic force measures 3.64 N, ensuring the stability of the levitation platform.
2. When the suspended platform was in the equilibrium position, the different bias currents ranging from 0.5 A to 1.4 A were applied to observe the change in electromagnetic force. Similarly, we set the equilibrium position at 0 mm and selected four sets of control currents to observe the change in electromagnetic force as the displacement varied from 0.1 mm to 0.4 mm. It was found that the slopes of the electromagnetic force curves remained relatively consistent. However, when the displacement exceeded 0.4 mm, the slope increased significantly, indicating the onset of electromagnetic force nonlinearity. These results suggest that the electromagnetic force exhibits a strong linear relationship within the equilibrium position range.
3. In the experiments, step signals were applied to the z, α, β, x, and y degrees of freedom. The experimental results indicate that the axial range is 0.05 mm, the radial range is 0.1 mm, and the range for the α and β degrees of freedom is 0.001 rad. Furthermore, sinusoidal signals were applied to the radial actuator, and the tracking characteristics were also analyzed, achieving the desired results in both cases.

In the future, our first priority will be to optimize the controller to address issues related to overshooting and response time. We plan to explore different control methods for regulating the five-degree-of-freedom magnetic levitation drive. Alternatively, we intend to integrate the 5-DOF magnetic levitation drive into a laser cutting head to investigate its impact on processing efficiency and quality under various control methods.

## Figures and Tables

**Table 1:** Theoretical calculation and simulation parameters.

Parameter Name	Symbol	Value
number of turns	N	100
Balance air gap	$d_{0}$ (mm)	1
permeability in vacuum	$\mu_{0}$	4π × 10^−7^
Air gap cross-sectional area	A (mm^2^)	60
Suspended platform inner diameter	$d_{1}$ (mm)	67
Suspended platform outer diameter	$d_{3}$ (mm)	114
Electromagnet cross section width	b (mm)	10
Electromagnet cross section length	$L_{4}$ (mm)	6
Coupling collar inner diameter	$d_{2}$ (mm)	126

**Table 2:** Model parameter.

Parameter Name	Symbol	Value
Quality	M (kg)	0.3
Moment of inertia about the X-axis	$J_{\alpha }$ (kg·m^2^)	9.7 × 10^−4^
Moment of inertia about the Y-axis	$J_{\beta }$ (kg·m^2^)	9.7 × 10^−4^
Sensor distance	$L_{2}$ (mm)	42.5
Axial current coefficient	$K_{i}$ (N/A)	1.13
Axial displacement coefficient	$K_{d}$ (N/m)	1.7 × 10^3^
Radial current coefficient	$K_{i}^{\prime }$ (N/A)	1.13
Radial displacement coefficient	$K_{d}^{\prime }$ (N/m)	1.7 × 10^3^
Damping coefficient in Z direction	$c_{z}$ (N/(m/s))	0.3
Damping coefficient in α direction	$c_{\beta }$ (N/(m/s))	9.7 × 10^−4^

## Data Availability

Data are contained within the article.
